# Change in compensated patient injuries in the treatment of Achilles tendon rupture: a nationwide analysis from 2000 to 2019

**DOI:** 10.2340/17453674.2025.44598

**Published:** 2025-10-03

**Authors:** Ida KIVIRANTA, Marjukka HALLINEN, Lauri KAUKONEN, Elina EKMAN, Joel KOSTENSALO, Päivi HELMIÖ, Heli KESKINEN

**Affiliations:** 1 Department of Orthopedics and Traumatology, Satasairaala Central Hospital, Pori; 2 Department of Orthopedics and Traumatology, Turku University Hospital, Turku, and the University of Turku, Turku; 3 Natural Resources Institute Finland, Natural Resources, Joensuu; 4 Department of Vascular Surgery, Turku University Hospital, Turku, and the University of Turku, Turku, Finland

## Abstract

**Background and purpose:**

Over the last 2 decades, the treatment of Achilles tendon rupture (ATR) has shifted from surgery to non-surgical methods. We aimed to analyze whether this change in treatment methods has had an impact on the number of compensated patient injuries in Finland and the grounds for compensation. We also aimed to investigate where injuries occur along the treatment pathway.

**Methods:**

We conducted a retrospective analysis of the Finnish Patient Insurance Centre’s insurance charts of compensated patient injuries in the treatment of ATR. Records of all compensated patient injury claims involving ATR from 2 periods in Finland: 2000–2006 (when 65% were treated surgically) and 2013–2019 (when 15% were treated surgically) were reviewed. Data included medical records, expert evaluations, and compensation decisions. Injuries were classified by when they occurred, and key contributing incidents were identified.

**Results:**

From 2000–2006 (period 1) and 2013–2019 (period 2), there were 315 patient injury claims related to ATR treatment in Finland. Of these, 126 (40%) were compensated. In both periods, delay in diagnosis was the most common reason for compensation. The number of claims remained the same between the 2 periods, and the ratio of compensated injuries to total cases declined (0.70% to 0.62%, not significant). Between the periods, infection-related claims decreased, while those related to incorrect treatment pathways and surgical errors increased (P = 0.02).

**Conclusion:**

The number of patient injuries has not risen in the past decade, while the number of infection injuries has decreased. Most patient injuries were related to a delay in diagnosis.

Acute Achilles tendon rupture (ATR) is a common injury among active middle-aged individuals, with increasing incidence rates, particularly among the elderly [1]. In Finland, the incidence increased from 17.3 to 32.3 per 100,000 person-years between 1997 and 2019, reflecting international trends in Western countries [2-4]. Irrespective of treatment choice, ATR can result in functional deficits and reduced activity levels [5,6]. 2 years after ATR, only 66% of patients considered their symptoms acceptable [7].

Timely diagnosis is critical, as delay in ATR diagnosis can close the timeframe for non-surgical treatment and lead to more complex surgery, with possible tissue reconstruction leading to an increased risk of postoperative infections and worse functional outcomes [5,8-11]. We know that operatively treated ATRs have been found to recover plantar flexion strength 10%–18% better than nonoperatively treated ATRs [12], and when considering delayed diagnosis in conservatively treated ATRs, it is hard to tell afterwards which part of the unsatisfactory outcome is due to the delay itself, and which is due to the treatment method.

In Finland, the Patient Insurance Centre (PIC) handles all patient injury-related claims related to medical treatment. In this study, claims compensated by PIC were considered as patient injuries, and this term is used throughout this article.

In the past 2 decades, surgical treatment for ATRs has decreased following scientific evidence supporting non-surgical treatment [13-15]. We aimed to analyze whether the change in treatment methods was associated with a change in the number of patient injuries and the grounds for compensation. We also aimed to investigate where injuries occur along the treatment pathway.

## Methods

### Study design

The study was a retrospective comparative analysis of Finnish national patient insurance charts of accepted and compensated patient injury claims concerning the treatment of ATRs.

Based on previous literature, we selected 2 distinct timeframes: the first represents a period when ATRs were treated mostly surgically and the second represents a period when ATRs were treated mostly non-surgically. We hypothesize that changes in treatment methods have altered the reasons for patient injuries between the 2 periods, with a relative increase in the proportion of injuries related to surgical treatment in the latter period.

The study is reported according to STROBE guidelines.

### Study data

Data was sourced by the PIC for 2 periods: January 1, 2000, to December 31, 2006, and January 1, 2013, to December 31, 2019. According to a previous study, during the first period in Finland, 65% of ATRs were treated surgically, whereas during the second period only 15% were treated surgically [16]. We included only closed claims classified under ICD-10 code S86.0 (Injury of Achilles tendon) and excluded claims that were not directly related to the treatment of ATR (e.g., problems with anesthesia during the surgery). We reviewed all medical records, expert evaluations, and compensation decisions related to the injury, and analyzed additional details concerning patients’ health status, healthcare providers, and healthcare institutions. In addition, information was gathered on surgeries, and duration and type of immobilization.

### Patient Insurance Centre in Finland

In Finland, the PIC handles all patient injury-related claims related to medical treatment. All care providers are mandated to obtain patient insurance under the Patient Injuries Act [17]. The insurance system is nationwide, covering both public and private healthcare providers. The PIC compensation system is patient driven, and claims must be submitted to the PIC within 3 years from when the patient became, or should have become, aware of the injury. As per the Patient Injuries Act (Potilasvahinkolaki 585/1986), there are 8 different reasons for compensation: treatment injury, infection injury, equipment-related injury, accident-related injury, fire-related injury, injury related to delivering pharmaceuticals, unreasonable injury, and implant injury. The majority of compensated injuries are classified as treatment injuries, with infection being the second most common reason for compensation [18]. In treatment injuries, the compensation criterion is that an experienced healthcare professional would have acted differently in the situation in question and thereby avoided the injury. Infections are compensable if deemed unexpected based on the patient’s health status and treatment. The other 6 reasons are rarely used. In some cases that are submitted to the PIC, the compensation criteria are met but the injury is deemed minor, and therefore no payment is made. In our data, these minor injuries are categorized as compensated injuries.

### Outcomes

#### Claims

The researchers examined the incidents and errors that contributed to the injury, and 1 to 3 key incidents were identified and classified. The structure of our classification was modified from the classification presented by Helmiö et al. [19]. Patient identities were excluded from the data.

#### Placement of injury

When comparing the placement of patient injuries along the treatment pathway, we defined injuries occurring before treatment as those that take place before the patient is fitted with a cast or brace or before surgery. Injuries during treatment were defined as injuries occurring during casting, bracing, or surgery, while other injuries were considered to have occurred after treatment.

### Statistics

To compare the claims submitted and the incidence of acute Achilles tendon ruptures, the incidence of ATRs (previously published by our research group [1]) was obtained from the National Hospital Discharge Register (NHDR) and the Finnish Register of Primary Health Care Visits (PHCR), both of which are maintained by the Finnish National Institute for Health and Welfare. The PHCR has been used since 2011 and, therefore, is analyzed only in the later timeframe.

For statistical testing, we considered the first claim submitted by the patient, as some patients had multiple claims. This was done to keep the observations independent, which is a crucial assumption of the statistical tests used in this study. While there were 47 second claims, 64% overlapped with the first claim, and only in 5 cases was the second claim approved while the first one was not.

The compensation reason was treated as a categorical variable, with compensation as a binary variable, while the incidence and number of claims were treated as count data. For the statistical significance of (i) the ratio of compensated insurance claims to the incidence of ATRs, (ii) the differences between the reasons for compensation, and (iii) the timing of the compensated claims along the treatment pathway we used Fisher’s exact test, as implemented in the stats package in R (R Foundation for Statistical Computing, Vienna, Austria). For the first test we used the exact version of the test, and for the latter 2 cases where the contingency tables were larger than 2×2, we used the approximate version of the test with 2,000 Monte Carlo simulations.

P value ≤ 0.05 was considered as statistically significant. All statistical analyses were carried out using the statistical software R version 4.4.2 [16].

### Ethics, funding, use of AI, and disclosures

The study protocol was approved by the PIC. Because this was a retrospective analysis of insurance charts, no separate ethics committee approval was needed for this study. The PIC, by law, provides researchers with the necessary materials, including injury-related patient charts, without requiring separate patient consent. The compensations paid for individual patient injuries in Finland are confidential and therefore cannot be published. AI (ChatGPT) has been used to streamline some parts of the text and to correct misspellings. All authors declare no conflict of interests. Complete disclosure of interest forms according to ICMJE are available on the article page, doi: 10.2340/17453674.2025.44598

## Results

**Figure 1:**
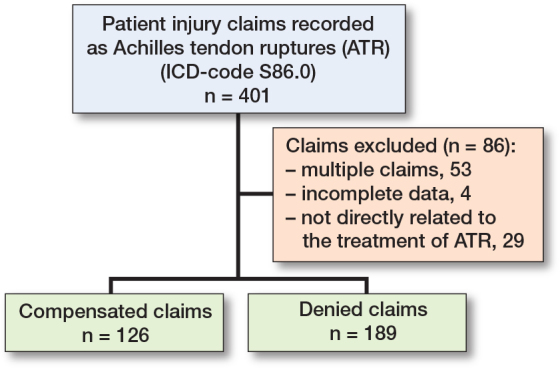
Flowchart of patient injury data collection.

**Figure 2:**
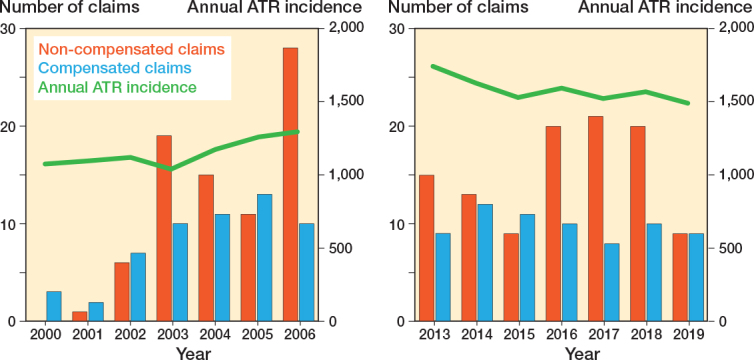
Number of ATR-related patient injury claims (red bar), approved compensated claims (blue bar), and annual ATR incidence (green line) by year (left panel: 2000–2006 and right panel: 2013–2019).

During the study periods 2000–2006 (period 1) and 2013–2019 (period 2), 315 patient injury claims were recorded regarding the treatment of Achilles tendon ruptures (Figure 1). The number of cases by year is presented in Figure 2. Altogether, cases from 126 patients were assessed as patient injuries and compensated corresponding to a rate of 40%. The mean age of the patients whose injury was compensated for was 55.0 years (SD 14, range 22–81) (Table 1), with 37% female, 28% smokers (among those with known smoking status), and 58% with an underlying health condition. There was no statistically significant difference between mean age and sex when comparing compensated and non-compensated claims. Of compensated claims, 86 patients underwent surgical treatment, of whom only 3 had a minimally invasive procedure.

**Table 1:** Number of compensated claims in 2000–2006 and 2013–2019 assigned to age groups

Age	2000–2006		2013–2019	
	Male	Female	Male	Female
20–39	10	4	8	3
40–59	15	8	11	14
60–69	12	3	15	6
≥70	3	1	2	6
Total	40	16	36	29

### Claims

The incidence of ATRs increased nationally during the study periods. Correspondingly, the number of patient injury claims increased from period 1 (n = 136) to period 2 (n = 178). There was no difference in ratio of compensated injuries between the 2 periods (56/8,007 = 0.70% during period 1, and 69/11,047 = 0.62% during period 2; P = 0.5).

There was a significant difference between the 2 periods regarding the reason for reimbursement (P = 0.02; Table 2). The number of reimbursed claims due to infection declined, while reimbursements for wrong treatment pathway and surgical error increased.

**Table 2:** Number of compensated claims in 2000–2006 and 2013–2019 categorized by reason and place along the treatment pathway. In period 1, there were no claims that included more than 1 reason for compensation. In period 2, 3 claims included 2 reasons for compensation

Reason for compensation in	n (%)	Beforetreatment	In treatment	After treatment
Period 1				
Delay in diagnosis	36 (64)	35	1	–
Infection	8 (14)	–	5	3
Re-rupture	0 (0.0)	–	–	–
Wrong treatment pathway	2 (3.6)	2	–	–
DVT	2 (3.6)	–	1	1
Surgical error	5 (8.9)	–	5	–
Cast- or brace-related error	3 (5.4)	–	3	–
Organizational error	2 (3.6)	2	–	–
Other	1 (1.8)	–	1	–
Period 2				
Delay in diagnosis	48 (70)	47	1	–
Infection	2 (2.9)	–	2	–
Re-rupture	0 (0.0)	–	–	–
Wrong treatment pathway	5 (7.2)	3	2	–
DVT	6 (8.7)	1	5	–
Surgical error	0 (0.0)	–	–	–
Cast- or brace-related error	3 (4.3)	1	2	–
Organizational error	0 (0.0)	–	–	–
Other	1 (1.4)	–	1	–

DVT: deep vein thrombosis.

### Placement of injury

When comparing where patient injuries occur along the treatment pathway, we found a significant difference between periods 1 and 2. The number of compensated claims before treatment was higher in period 2 than in period 1 (P = 0.03) (see Table 2).

## Discussion

This is the first study on the incidence or types of patient-reported injury claims related to ATRs. We showed that even though the incidence of ATRs has risen in the past 2 decades, no evidence of a change in the proportion of compensated claims was found, but the proportion of compensated infection injuries was clinically smaller during the latter period. The number of compensated claims before treatment was higher in the latter period than in the first period.

Patient injuries related to the treatment of ATRs are rare, but the consequences for patients can be severe. In our study, the incidence of ATR-related patient injuries in Finland was 0.66%, and 40% of the reported claims were compensated. In 2024, 21% of all reported claims to the PIC were compensated [20]. In comparison, the compensation percentage in our study is relatively high. It is notable that the majority of compensated patient injuries are related to surgical procedures on the musculoskeletal system, and the compensation percentage in these procedures can be greater than in claims in general [20]. The compensation rate in our study is consistent with previous analyses of the PIC register concerning other orthopedic injuries: compensation rates of 40% for ACL-related injuries [21] and 36% for claims related to distal radius fractures [22]. A Danish study on ATR-related patient claims reported a compensation rate of 41%, closely mirroring the findings of our study [23].

The number of claims, compensated or not, did not increase between the 2 periods. Unlike ATRs, in Finland the number of all reported claims, regardless of specialty, has shown a long-term increase [18,24,25]. This may be due, in part, to increased public awareness of patient insurance and the ability to file claims easily online. It remains unclear, however, why the number of claims concerning ATRs has not increased in the same way as it has with other claims. It is possible that the change in treatment method toward non-surgical remedies has already shown benefits by decreasing the patient claims related to ATRs.

We found that between 2013 and 2019, the number of compensated infection injuries dropped from 14% to 2.9%. The literature indicates that the risk of wound healing issues after ATR surgery ranges from 1.4% to 10%, while infection risk in non-surgically treated ATRs is negligible [26-28]. Although the risk of infection after surgery is low, the consequences for the patient can be severe.

In our study, the most common reason for compensation was a delay in diagnosis. This finding is similar to a Danish study in which 35% of all reported patient injury claims concerned overlooked diagnoses, with 50% of them compensated [23]. Diagnostic delays are found to be more common in elderly patients with atypical injury mechanisms [29]. An American study by Raikin et al. found that 32% of ATRs in people over 55 years of age were diagnosed with a delay of longer than 4 weeks, in accordance with our study [30]. Another study found that of 15,045 patients who underwent surgery for ATR, the primary diagnosis was wrong in 6.6% of cases, which means that the initial injury diagnosis they received was changed to an ATR diagnosis at a subsequent visit within 14 days [31].

When comparing the 2 timeframes, we found that the number of claims concerning casting or bracing was higher in the first timeframe and decreased in the second timeframe. It seems that the increased number of non-surgically treated patients, and therefore the increased number of castings, has led to a decreased number of claims concerning cast or brace immobilization, which may be explained by better skills due to handling of more casts.

### Strength

The strength of this study is its highly representative and nationwide scope, as the PIC insures all patients, processes all claims, and provides compensation in Finland.

### Limitations

It is notable that our study population was highly selective and included only patients who later filed claims concerning diagnostic errors. While healthcare professionals are required by law to inform patients about submitting injury claims, not all injuries may be reported. The PIC registers only claims submitted by patients; therefore, the actual number of injuries may be higher than has reported in this study. Also, there is always a risk of incorrect ICD-code registration, which may have resulted in some claims being missed. As this study design was a retrospective analysis, we were dependent on the data recorded in charts, which could have resulted in inaccurate or missing data for some patients.

### Conclusion

The number of patient injuries has not risen in the past decade, while the number of infection injuries has decreased. Most patient injuries were related to a delay in diagnosis.

*In perspective,* to prevent future patient injuries, more focus should be placed on early diagnosis of ATR. Physicians must consistently consider ATR in the differential diagnosis, particularly in elderly patients or those with atypical injury mechanisms.

## Notes

All authors have accepted responsibility for the entire content of this manuscript and consented to its submission to the journal, reviewed all the results and approved the final version of the manuscript. EE, PH, and HK: conceived the idea and supervised the project. IK and LK: collected the data from PIC charts. JK: formal analysis, methodology, visualization. IK and MH: wrote the manuscript in consultation with EE, HK, and PH. All authors discussed the results and contributed to the final manuscript.

Handling co-editor: Per Henrik Randsborg

*Acta* thanks Tommy Frøseth Aae and Kristoffer Weisskirchner Barfod for help with peer review of this manuscript.
